# Arsenic Induces Continuous Inflammation and Regulates Th1/Th2/Th17/Treg Balance in Liver and Kidney In Vivo

**DOI:** 10.1155/2022/8414047

**Published:** 2022-02-15

**Authors:** Xiaoxu Duan, Guowei Xu, Jinlong Li, Nan Yan, Xin Li, Xuping Liu, Bing Li

**Affiliations:** ^1^Environment and Non-Communicable Disease Research Center, Key Laboratory of Arsenic-Related Biological Effects and Prevention and Treatment in Liaoning Province, School of Public Health, China Medical University, Shenyang 110122, China; ^2^Department of Toxicology, School of Public Heath, Shenyang Medical College, Shenyang, 110034 Liaoning Province, China; ^3^Department of Occupational and Environmental Health, Key Laboratory of Occupational Health and Safety for Coal Industry in Hebei Province, School of Public Health, North China University of Science and Technology, Tangshan 063210, China

## Abstract

Numerous studies on arsenic-induced hepatonephric toxicity including cancer have been reported. Given that chronic inflammatory response and immune imbalance are associated with oncogenesis, we investigated whether arsenic could influence the hepatic and nephritic expression of inflammatory factors and the differentiation of T cells. Mice were exposed to NaAsO_2_ (0, 25, and 50 mg/L) for 1 and 3 months. Our data showed the destruction of the structure and inflammatory infiltration in the liver. The arsenic markedly increased the activity of serum alanine aminotransferase (ALT) and aspartate aminotransferase (AST). The myeloperoxidase (MPO) activities increased in the liver at 25 and 50 mg/L arsenic for 3 months as well as in the kidney at both 1 and 3 months. An increased expression of inflammatory indicators (IL-1*β*, IL-12, and TNF-*α*) at 25 and 50 mg/L arsenic for 1 and 3 months in the liver and kidney, as well as IL-1*β* in the liver for 3 months and in the kidney at 50 mg/L for 1 and 3 months were demonstrated in our experiments. Besides, a definite tendency toward Th1/Th17 cytokines in the liver while Th2/Th17 cytokines in kidney was also observed by arsenic. Moreover, arsenic enhanced the expression of MAPK/Nrf2/NF-*κ*B signaling molecules. In conclusion, the results of the study suggested that arsenic induces continuous immune-inflammatory responses in the liver and kidney.

## 1. Introduction

Arsenic is the most hazardous compound in the environment, which continues to be a major global health problem worldwide [1]. Chronic arsenic exposure could induce many cancers including skin, bladder, and lung, as well as noncancer diseases, such as diabetes, cardiovascular diseases, and anemia [2, 3]. The liver and kidney are the major target organs for arsenic poisoning since they play the important roles in arsenic metabolism and excretion, respectively [4]. Increasing studies have found that chronic arsenic exposure could cause liver injury, hepatoportal sclerosis, and liver cancer [5, 6]. Toxicological studies also have documented that arsenic exposure could induce renal edema and inflammatory infiltration and result in acute renal failure including nephritis, nephritic syndrome, and nephrosis [7]. Despite the knowledge advancements from studies of arsenic exposure on the hepatotoxicity and nephrotoxicity underlying in vivo, a detailed molecular mechanism has not yet been well understood.

Except for its carcinogenicity, arsenic also has immunotoxicity. CD4^+^ T cells play a crucial role in regulating the immunity, inflammation, and cancer [8]. Animal studies have revealed that arsenic exposure affected CD4^+^ T cell numbers and CD4/CD8 ratios in the spleen and thymus [9, 10]. An epidemiologic study found that the maternal urinary arsenic concentrations were negatively associated with CD45RA+ CD4^+^ cells in cord blood [11]. CD4+ T cells could differentiate into Th1, Th2, Th17, and Treg by a series of corresponding transcription factors and cytokines [12]. It has been reported that arsenic has immunesuppressive effect destroying Th1/Th2 imbalance [13], inhibiting Th17 cell differentiation, and promoting regulatory T (Treg) cell generation [14]. On the other hand, the liver and kidney were considered to possess macrophages, DCs, and other immune cells, which may also be involved in balancing immunity and tolerance [15, 16]. Growing evidences have indicated that chronic inflammation could continuously produce reactive oxygen species (ROS) and NO, which may cause cell damage and in turn induce cell proliferation, leading to DNA damage and gene mutation, and finally to the occurrence and development of tumor [17]. In addition, researchers also have proposed that the reduced immune surveillance could trigger different types of diseases including malignancies [18]. However, the study on the comprehensive immune-inflammatory response and potential mechanism by arsenic has not been reported so much.

The main mechanism for arsenic toxicity was the ROS generation and therefore the induction of oxidative stress [19]. In addition, excess ROS could trigger the activation of mitogen-activated protein kinase (MAPK) and nuclear factor E2-related factor 2 (Nrf2) signal pathways, which were involved in promoting and suppressing carcinogenesis [20]. It has been also reported that arsenic could activate the MAPK and Nrf2 pathway, then regulate the expression of inflammatory mediators including IL-1*β*, TNF-*α*, IL-6, and IL-12, and result in neural inflammation and autophagy [21]. However, the knowledge on the effects of subchronic arsenic exposure on the hepatic and renal MAPK and Nrf2 pathways is still in initial stage.

Therefore, the study intended to investigate the immune dysfunction and inflammatory response in the liver and kidney by examining subchronic arsenic exposure model, hepatic and renal pathological and biochemical index alteration, the expression of inflammation (IL-1*β*, IL-6, IL-12, and TNF-*α*), and the markers representing the T cell differentiation in the liver and kidney. Moreover, we also surveyed the relevant mechanism by observing the related immune-inflammatory modulatory pathway MAPK, nuclear factor kappa B (NF-*κ*B), and Nrf2. We are trying to provide a novel insight for understanding the carcinogenic mechanism of arsenic in the liver and kidney.

## 2. Materials and Methods

### 2.1. Reagents and Chemicals

Sodium arsenite (≥99.0%) was obtained from Sigma Chemical Co. (St. Louis, MO, USA). Myeloperoxidase (MPO), alanine aminotransferase (ALT), and aspartate aminotransferase (AST) detection kits were purchased from Jiancheng Biological Institute (Nanjing, China). Real-time polymerase chain reaction (real-time PCR) kits were from Takara Co. (Otsu Japan). Primary antibodies of ERK1/2, P-ERK1/2, JNK, P-JNK, P38, and P-P38 were purchased from Cell Signaling Technology (Cell Signaling, Danvers, USA), and NF-*κ*B, Nrf2, GSTO1/2, heme oxygenase 1, *β*-actin, and corresponding secondary antibodies were all purchased from Santa Cruz Biotechnology (Santa Cruz, CA, USA). All other chemicals used were of analytical grade.

### 2.2. Animals and Experimental Procedures

Six-week-old female Kunming mice of 18–22 g were obtained from the Center for Experimental Animals at China Medical University (Shenyang, China) with a National Animals Use License number of SCXK-LN2013-0007. Mice were group-housed in stainless steel cages (10 mice per cage) in an air-conditioned room with temperature maintained at 22 ± 2°C and 12 h light/dark cycle for 1 week before the experiment. The mice were allowed standard mice chow diet and drinking water ad libitum throughout the study. All experiments and surgical procedures were approved by the Animal Care and Use Committee of China Medical University.

The dose for NaAsO_2_ was selected on the basis of previously published studies [22], as well as our preliminary experiments. Mice were exposed to NaAsO_2_ in drinking water at concentrations of 0, 25, and 50 mg/L for 1 and 3 months. The food and water consumption were measured every three days, and the mice were weighed every week during the experimental period. At the end day of the experiment, all mice were weighed and deeply anesthetized. Blood was collected through eyeball, extirpating into heparinized vials and centrifuged (3000x g, 4°C) for 10 min; the serum obtained was kept frozen at −80°C for measure. The entire liver and kidney were promptly removed and weighed, and small liver and kidney fractions were fixed with 4% paraformaldehyde for histopathological studies, and the remaining tissues were stored at −80°C for biochemical use.

### 2.3. Determination of Total Arsenic Concentration in Liver and Kidney

Measurement of arsenic species was performed as described by Li et al. [23]. Briefly, the liver and kidney was homogenized on ice with 10 ml deionized water per gram of tissue weight. iAs, monomethylarsonic acid (MMA), and dimethylarsinic acid (DMA) were determined by a high-performance liquid chromatography-hydride generation-atomic fluorescence spectrometer (HPLC-HG-AFS, SA-10 Atomic Fluorescence Species Analyzer, Titan Co., Beijing). Total arsenic (T-As) levels in the liver and kidney were then calculated by summing up the levels of iAs, MMA, and DMA totally.

### 2.4. Histopathological Analysis

Histopathological evaluations of the liver and kidney were performed according to the standard laboratory procedures. Briefly, the liver and kidney from three mice were removed and fixed with 4% paraformaldehyde for 48 h and embedded in paraffin blocks. 5 *μ*m sections were prepared by microtome (EM UC7, Leica, Germany) and then stained for 15 min with hematoxylin-eosin (H&E) (Solarbio, BeiJing, China), mounted, and analyzed using optical microscopy with a digital imaging system (Biodirect-Inc., Nikon, Japan). The injury score of the liver was evaluated as described by Kleiner et al. [24].

### 2.5. Determination of Serum Aminotransferase and Hepatonephric MPO Activities

ALT and AST in the serum, as well as MPO in the liver and kidney, were measured using commercially available kits according to the manufacturer's instructions.

### 2.6. Total RNA Isolation and Real-Time PCR Analysis

Total RNAs of the liver and kidney were isolated using a TRIzol reagent (Invitrogen, USA). 500 ng RNA was reversely transcribed into cDNA and amplified by using Takara reagent (Takara, Japan) according to the manufacture′s protocol; then, PCR amplification was performed by SYBR Premix ExTaqII kits (Takara, Japan). PCR was performed using the following thermal cycling conditions: 95°C 30 s; 40 cycles of denaturing at 95°C for 5 s; and annealing at 60°C for 30 s. PCR was performed using the following primers: IL-1*β* (F): TGACCTGGGCTGTCCTGATG, (R): GGTGCTCATGTCCTCATCCTG, product length: 220 bp; IL-6 (F): CTGCAAGAGACTTCCATCCAG, (R): AGTGGT ATAGACAGGTCTGTTG, product length: 131 bp; IL-12 (F): TGGTTTGCCATCGTTTTGCTG, (R): ACAGGTGAGGTTCACTGTTTCT, product length:123 bp; Tnf-*α* (F): CCCCAAAGGGATGAGAAGTTC, (R): GGCTTGTCACTCGAATTTTGAGA, product length: 148 bp; Ifn-*γ* (F): AAGCGTCATTGAATCACACCTG, (R): TGAC CTCAAACTTGGCAATACTC, product length: 92 bp; IL-13 (F): CACACAAGACCAGACTCCCCTG, (R): GGTTACAGAGGCCATGCAATATCC, product length: 155 bp; IL-23 (F): CCCGTATCCAGTGTGAAGATG, (R): CCCTTTGAAGATGTCAGAGTC, product length: 128 bp; IL-10 (F): GGGGCCAGTACAGCCGGGAAA, (R): CTGGCT GAAGGCAGTCCGCA, product length: 92 bp; GADPH (F): TGTGTCCGTCGTGGATCTGA, (R): TTGCTGTTGAAGTCGCAGGAG, product length: 150 bp. 2^−*ΔΔ*Ct^ values were calculated to represent the amounts of different target genes.

### 2.7. Western Blot Analysis

The total proteins of the liver and kidney were extracted by commercial kits, and protein concentrations were quantified by bicinchoninic acid (BCA) protein kit (Beyotime, Shanghai, China). 45 *μ*g total protein was boiled for 5 min at 100°C before 7.5–10% SDS-PAGE and then transferred to 0.45 *μ*M polyvinylidene fluoride (PVDF) membrane (Amersham, Buckinghamshire, UK). After blocking for 2 h at room temperature, membranes were then probed with the primary antibodies of ERK1/2, P-ERK1/2, JNK, P-JNK, P38, P-P38, NF-*κ*B, Nrf2, GSTO1/2, and HO-1 (1 : 1000) at 4°C overnight, respectively. Finally, membranes were incubated with corresponding secondary antibodies (1–5000) for 2 h at room temperature. Blots were detected with chemiluminescence reagents (PicoWest Super Signal, Pierce Biotechnology, IL, USA) and visualized using Electrophoresis Gel Imaging Analysis System (MF-ChemiBIS 3.2, DNR Bio-Imaging Systems, Israel). *β*-Actin (1 : 5000) was used as the internal control.

### 2.8. Statistical Analysis

Data were expressed as mean ± standard deviation (SD). Comparisons among groups were made using one-way analysis of variance (ANOVA) with LSD post hoc test using the SPSS 25.0 statistical analysis software. *P* < 0.05 was considered to be statistically significant.

## 3. Results

### 3.1. General Status of Study Mice

In our study, mice were treated with 25 and 50 mg/L NaAsO_2_ for 1 and 3 months, respectively, by drinking water. All animals survived to the end of the experiment. The calculated average daily arsenic intake of different treatment groups was listed in Table 1. T-As levels in the liver and kidney were increased dramatically by arsenic exposure. We found that the weight of the liver significantly decreased in NaAsO_2_-treated mice compared with corresponding control mice (*P* < 0.05), while no changes as to the kidney among different treatments. No statistically significant differences of body weight, as well as general status, have been observed during the whole study.

### 3.2. Subchronic Arsenic Exposure Induces Tissue Histopathology and Dysfunction of Liver and Kidney

**Figure 1:**
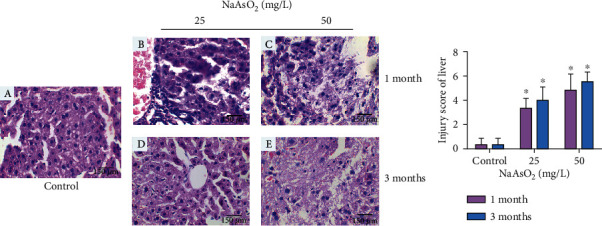
Histopathological changes in hepatic tissues of subchronic arsenic-treated mice by hematoxylin-eosin (H&E) assay (original magnification: ×400). Mice were treated with 25 and 50 mg/L NaAsO_2_ for 1 and 3 months: (a) Control; (b) 25 mg/L NaAsO_2_-treated mice for 1 month; (c) 50 mg/L NaAsO_2_-treated mice for 1 month; (d) 25 mg/L NaAsO_2_-treated mice for 3 months; and (e) 50 mg/L NaAsO_2_-treated mice for 3 months. (f) Analytical result of the liver injury score. Data were expressed as mean ± SD. ^∗^*P* < 0.05 compared with control mice.

We performed HE stain to investigate the histopathologic changes in the liver and kidney. As shown in Figure 1, the histological profile of the liver showed a normal hepatic architecture with hepatic lobules and hepatocytes in the control group (Figure 1(a)). Compared with the control group, the animals administered with 25 mg/L NaAsO_2_ showed obvious inflammatory cell infiltration at 1 month (Figure 1(b)). With the increase of dose and time, we observed subsequently extensive disruption of the liver architecture including hepatocellular necrosis (Figures 1(c)–1(e)). However, we failed to find evident changes in the kidney of arsenic-treated groups (data not shown).

**Figure 2:**
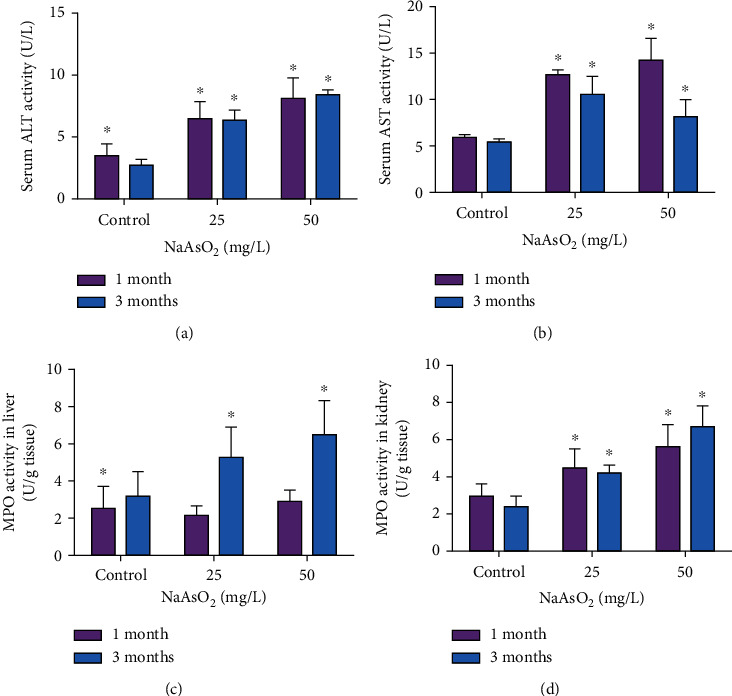
Subchronic arsenic exposure increased the activities of serum alanine amino transferase (ALT) and aspartate aminotransferase (AST), as well as hepatorenal myeloperoxidase (MPO) in mice. Mice were treated with 25 and 50 mg/L NaAsO_2_ for 1 and 3 months. The activities of serum ALT (a) and AST (b) and MPO in the liver (c) and kidney (d) were determined. Data were expressed as mean ± SD. ^∗^*P* < 0.05 compared with control mice.

ALT and AST are the most common biochemical indexes of hepatic injuries. In our results, serum ALT enzyme activity upregulated dramatically with a dose-effect relationship by 25 and 50 mg/L NaAsO_2_ exposure at 1 and 3 months (Figure 2(a)). Likewise, our results also verified the clear increase of AST enzyme activity in serum by arsenic exposure comparing with the control group (Figure 2(b), *P* < 0.05)

Our pathological results showed that arsenic induced marked inflammatory cell infiltration; we further evaluated inflammation-associated infiltration of neutrophils and monocytes within the tissue by measuring hepatic and renal MPO activity. The MPO activity in liver was increased by 65% and 105% at 25 and 50 mg/L NaAsO_2_ in 3 months (Figure 2(c), *P* < 0.05). By contrast, we also found a notable enhancement of renal MPO activity at 25 and 50 mg/L NaAsO_2_ in both 1 and 3 months (Figure 2(d), *P* < 0.05). These changes indicated that subchronic arsenic exposure could induce tissue damage and inflammation in mice.

### 3.3. Subchronic Arsenic Exposure Increases the Expression of Inflammatory Cytokines in Liver and Kidney

**Figure 3:**
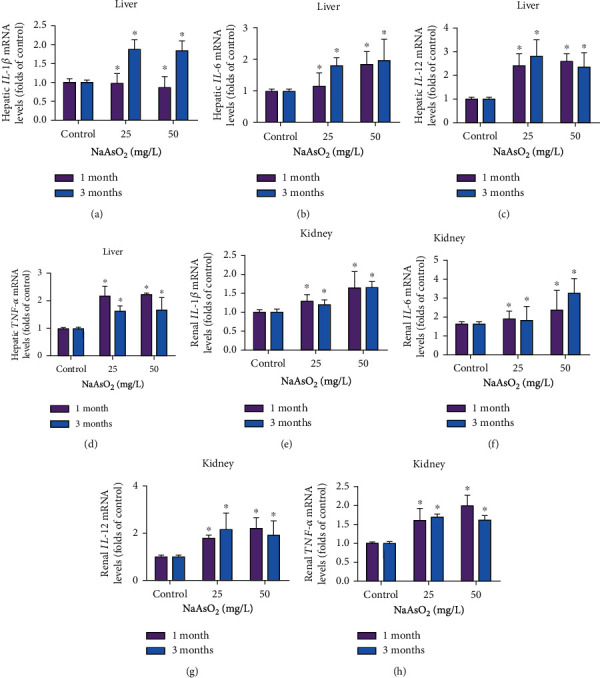
Subchronic arsenic exposure raised the levels of inflammatory cytokines in the liver and kidney. Mice were treated with 25 and 50 mg/L NaAsO_2_ by drinking water for 1 and 3 months. The mRNA levels of IL-1*β*, IL-6, IL-12, and TNF-*α* in liver (a–d), as well as in kidney (e–h) were determined by real-time PCR. Data were expressed as mean ± SD. ^∗^*P* < 0.05 compared with control mice.

Inflammatory cytokines IL-1*β*, IL-6, IL-12, and TNF-*α* in the liver and kidney were determined by real-time PCR. The mRNA levels of hepatic IL-12 and TNF-*α* were all elevated markedly by 25 and 50 mg/L NaAsO_2_ for 1 month (Figures 3(c) and 3(d), *P* < 0.05), 50 mg/L arsenic also enhanced the IL-6 mRNA levels in the liver (Figure 3(b), *P* < 0.05). In addition, the mRNA levels of hepatic IL-1*β*, IL-6, IL-12, and TNF-*α* were all elevated markedly in the arsenic-treated group at 3 months (Figures 3(a)–3(d), *P* < 0.05). By contrast, we also found a notable enhancement of IL-6, IL-12, and TNF-*α* in different arsenic-treated groups, as well as a little increase of IL-1*β* mRNA levels in the kidney by 50 mg/L NaAsO_2_ exposure for 1 and 3 months (Figures 3(e)–3(h), *P* < 0.05). These results indicated that subchronic arsenic exposure could affect the expression of inflammatory cytokines and induce the overall inflammatory response in both the liver and kidney.

### 3.4. Subchronic Arsenic Exposure Affects the Differentiation of CD4^+^ T Cell of Liver and Kidney

**Figure 4:**
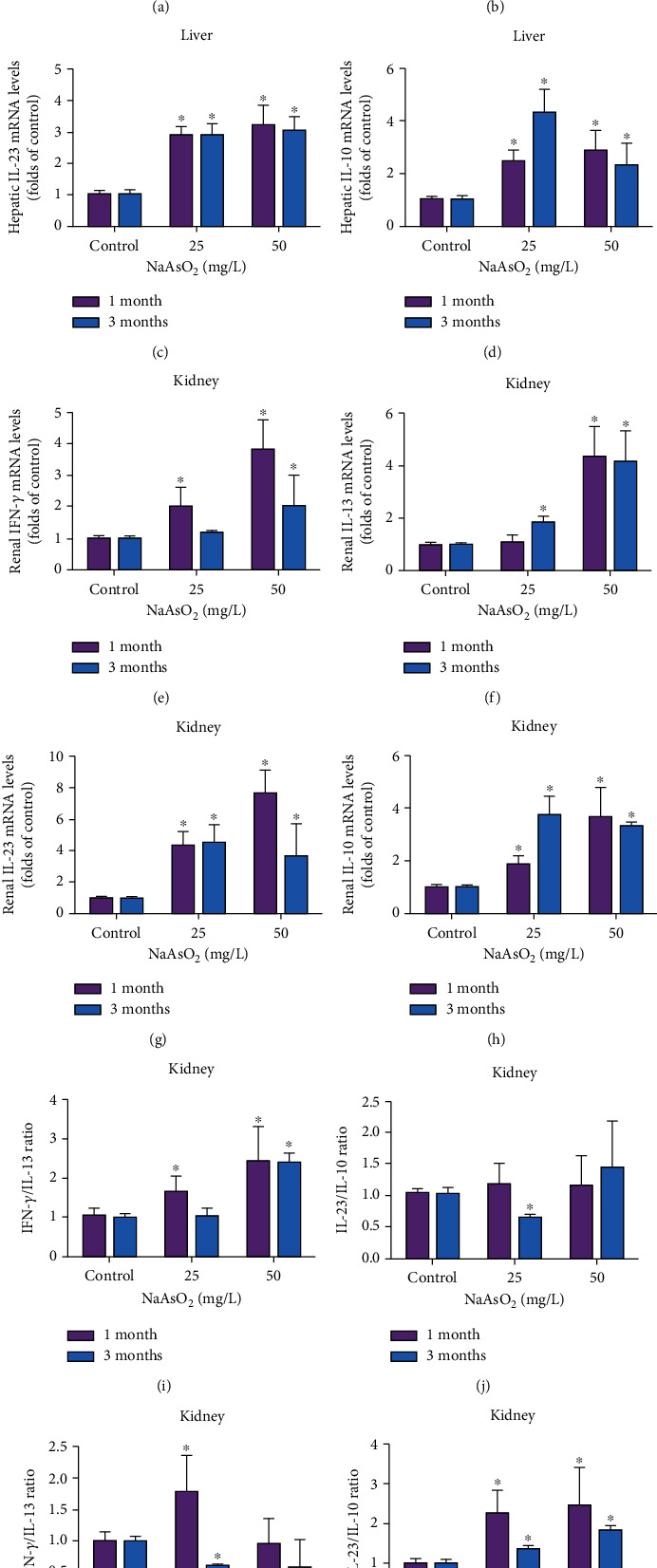
Suchronic arsenic exposure affected the expression of CD4^+^ T cell-specific signature cytokines in the liver and kidney. Mice were treated with 25 and 50 mg/L NaAsO_2_ by drinking water for 1 and 3 months. The mRNA levels of T cell signature cytokines IFN-*γ*, IL-13, IL-23, and IL-10 in the liver (a–d) and kidney (e–h) were determined by real-time PCR; the ratios of IFN-*γ*/IL-13 and IL-23/IL-10 were calculated in liver (i–j) and kidney (k–l). Data were expressed as mean ± SD. ^∗^*P* < 0.05 compared with control mice.

It was reported that the liver and kidney also include CD4^+^ T cell, macrophagocyte, and dendritic cells, which play important roles in maintaining immune homeostasis. Next, we measured the levels of mRNA encoding T helper 1 (Th1), Th2, Th17, and regulatory T cells- (Treg cell-) specific cytokines. Th1 cytokine IFN-*γ*, Th2 cytokine IL-13, Th17 cytokine IL-23, and Treg cytokine IL-10 mRNA levels all increased dramatically in the liver at arsenic-treated groups (Figures 4(a)–4(d), *P* < 0.05). By contrast, the levels of renal Th1 cytokine IFN-*γ* mRNA markedly increased by 100% and 282% at 25 mg/L NaAsO_2_ with 1 and 3 months, 101% at 50 mg/L NaAsO_2_ with 3 months (Figure 4(e), *P* < 0.05), while Th2 cytokine IL-13 mRNA levels were upregulated by 85% at 25 mg/L NaAsO_2_ with 3 months, 334% and 315% at 50 mg/L NaAsO_2_ with 1 and 3 months in the kidney (Figure 4(f), *P* < 0.05). We also found a notable enhancement of Th17 cytokine IL-23 and Treg cytokine IL-10 in the kidney of different arsenic groups (Figures 4(g) and 4(h), *P* < 0.05).

We also calculated the ratios of Th1/Th2 and Th17/Treg cytokines in the liver and kidney to more accurately reflect the effect of subchronic arsenic exposure on CD4^+^ T cell differentiation. The results showed that compared with the control group, hepatic IFN-*γ*/IL-13 ratio and IL-23/IL-10 ratio were increased at 25 mg/L NaAsO_2_-treated group with 1 and 3 months and at 50 mg/L NaAsO_2_-treated groups with 3 months (Figures 4(i) and 4(j), *P* < 0.05), while IL-23/IL-10 ratio decreased a little at the 25 mg/L NaAsO_2_-treated group with 3 months (Figure 4(j), *P* < 0.05). In the kidney, IFN-*γ*/IL-13 ratio was upregulated at the 25 mg/L NaAsO_2_-treated group with 1 month and decreased at the 25 mg/L NaAsO_2_-treated group with 3 months as well as 50 mg/L NaAsO_2_-treated groups with 1 and 3 months (Figure 4(k), *P* < 0.05). The ratio of IL-23/IL-10 in the kidney was increased in all arsenic-treated groups (Figure 4(l), *P* < 0.05). These results indicated that subchronic arsenic exposure could affect the expression of CD4^+^ T cell subpopulation-related cytokines and induce a prominent advantage of Th1 and Th17 in the liver but Th2 and Th17 in the kidney.

### 3.5. Subchronic Arsenic Exposure Activates MAPK/NF-*κ*B/Nrf2 Pathway of Liver and Kidney

**Figure 5:**
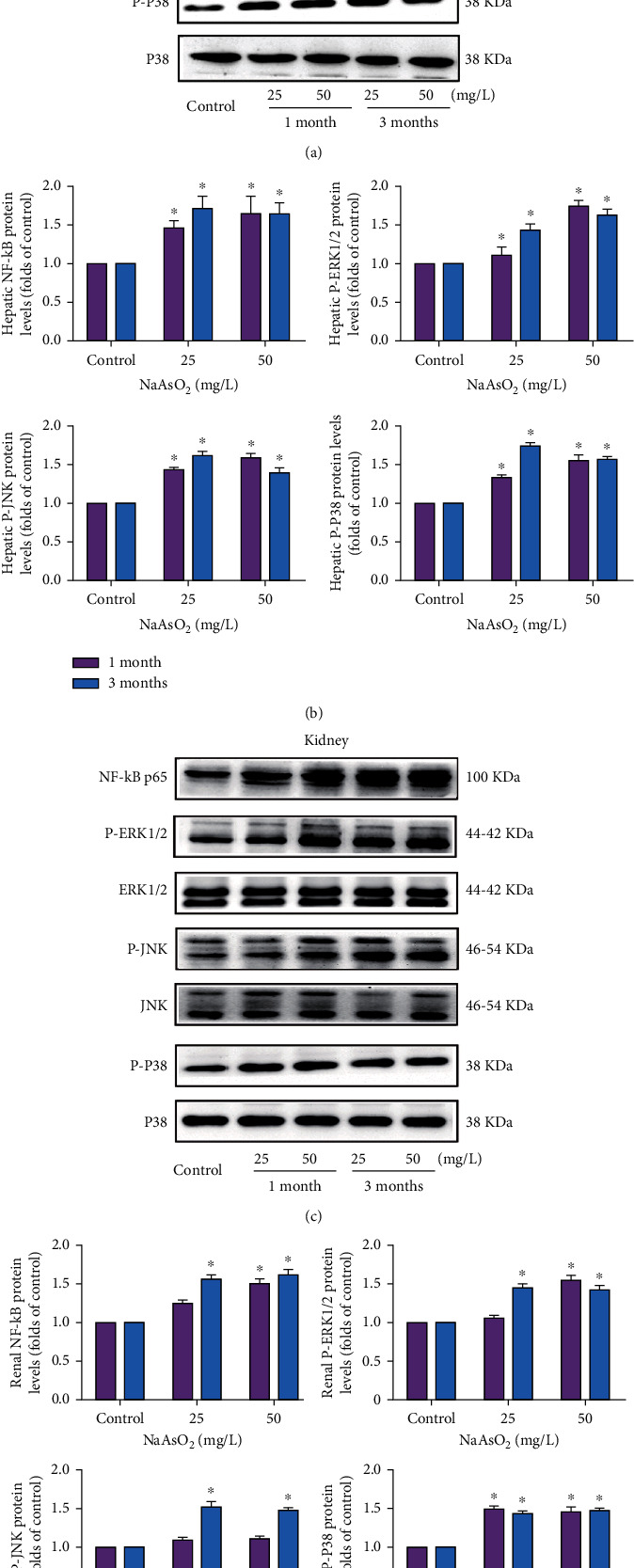
Subchronic arsenic exposure activated MAPKs/NF-*κ*B in the liver and kidney. Mice were treated with 25 and 50 mg/L NaAsO_2_ by drinking water for 1 and 3 months. Expression of NF-*κ*B, P-ERK1/2, P-JNK, and P-P38 in the liver (a) and kidney (c), and the corresponding quantitative analysis (b, d) were assessed by western blotting; *β*-actin was blotted as the loading control. Data were expressed as mean ± SD. ^∗^*P* < 0.05 compared with control mice.

**Figure 6:**
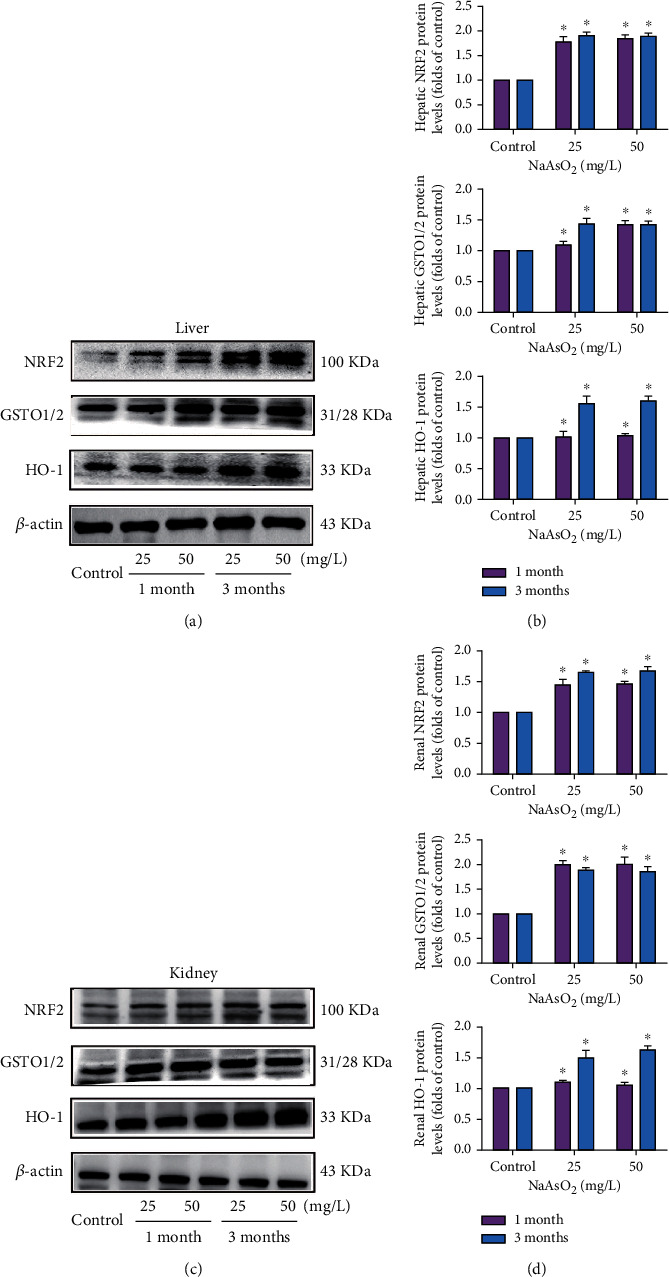
Subchronic arsenic exposure activated the Nrf2 pathway in the liver and kidney. Mice were treated with 25 and 50 mg/L NaAsO_2_ by drinking water for 1 and 3 months. Expression of Nrf2, GSTO1/2, and HO-1 in the liver (a) and kidney (c) and the corresponding quantitative analysis (b, d) were assessed by western blotting; *β*-actin was blotted as the loading control. Data were expressed as mean ± SD. ^∗^*P* < 0.05 compared with control mice.

We observed that phosphorylations of ERK1/2, JNK, and P38 were significantly induced by arsenic in the liver and kidney (Figure 5). Moreover, the expression of NF-*κ*B protein was markedly upregulated 1.5–2 times of control in the liver by arsenic (Figure 5(a)). The upregulation of renal NF-*κ*B protein was consistent with the clear increase of the liver (Figure 5(c), *P* < 0.05). As shown in Figures 6(a) and 6(c), arsenic treatment showed a clear increase of Nrf2 and GST protein in the liver and kidney (*P* < 0.05). The antioxidant enzyme HO-1 was elevated markedly in the 25 and 50 mg/L NaAsO_2_ groups with 3 months, and the upregulation of renal HO-1 protein was consistent with the clear increase of liver. Totally, the subchronic arsenic exposure induced the continuous activation of the MAPK/NF-*κ*B/Nrf2 pathway, which might also be associated with the arsenic-induced immune-inflammatory imbalance response.

## 4. Discussion

Arsenic is well known to cause numerous cancers including the liver and kidney; mechanistic studies have also reported that it may be associated with genotoxicity-related DNA methylation, oxidative stress, and altered cell proliferation. Recent years, the critical roles of decreased immune surveillance and chronic inflammatory response in the occurrence and development of cancer have attracted great interest. So, we investigated the effect of subchronic arsenic exposure on hepatic and renal immune-inflammatory responses as well as the underlying mechanism in a mouse model.

Histopathological examination is viewed as the “gold standard” to determine organ damage. One year of 6 *μ*g/gm arsenic body weight/day feed induced the pathologic changes including mild hepatic steatosis, inflammation, necrosis, and significant fibrosis in mice [25]. Our results were corroborated by the similar findings of the liver. In another study, Xu et al. observed early glomerular sclerosis, tubular atrophy, and marked chronic inflammatory cell infiltration of the kidney interstitial space in the high-arsenic treatment group [26]. However, there were no obvious renal pathological changes in our results. Based on the important role of infiltration of neutrophils and monocytes in inflammatory response, we analyzed hepatic and renal MPO activities which have been used as a reliable marker of tissue inflammation. The significant increase in MPO activities of the liver and kidneys tissue after arsenic exposure in our study is consistent with other studies [27]. We also tested the ALT and AST activities in the serum, which are the important indicators for liver damage and immune response. Many studies have reported that the activities of ALT and AST in the plasma were significantly higher in arsenic-treated rats than in normal control rats [28]. Consistent with these studies, we observed markedly raised levels of serum ALT and AST in arsenic-treated mice; it may be associated with arsenic-induced hepatocyte membrane damage.

Inflammatory cell infiltration could increase secretion of various inflammatory cytokines and thus impair immune function [29]. In addition, chronic inflammatory response has been found to be one of the most important factors that contribute to cancers [30]. It was reported that proinflammatory cytokines TNF-*α*, IL-6, and IL-1*β* are crucial to immune response, inflammatory, and hematopoiesis, as well as development and progression of tumor [17]. Animal studies have determined that arsenic exposure stimulates proinflammatory cytokine TNF-*α*, IL-6, and IL-8 gene expressions in the liver of the cocks [31]. In another study, arsenic reportedly stimulated the expression of inflammatory gene iNOS, cyclooxygenase2 (COX-2) and TNF-*α* protein, and mRNA in the kidney of chicken [32]. Our experimental results showed that arsenic significantly increased the levels of hepatonephric IL-1*β*, IL-6, Il-12, and TNF-*α* in both 1 and 3 months, suggesting that subchronic arsenic exposure could lead to hepatorenal persistent inflammatory response, which may be further meaningful to raise the incidences of liver and kidney cancers.

Many studies have reported that arsenic could regulate Th1 and Th2 response by affecting the section of their representative cytokines such as IFN-*γ*, IL-4, and IL-13 [32, 33]. Th17 has strong plasticity and can be transformed into Tregs and Tr1 (Type I regulatory T cells) under pathological conditions, therefore exerting immunosuppressive effects [25]. IL-13 and IL-23 are essential for the differentiation of Th2 as well as Th17 [34, 35]. In addition, IL-23 is found to be overexpressed in many human cancers as well as mouse tumors [34]. Treg cells, which are characterized by IL-10 production, can modulate Th1, Th2, and Th17 immune responses via multiple mechanisms [36]. Our results showed that subchronic arsenic exposure raised IFN-*γ*, IL-13, IL-23, and IL-10 in the liver and kidney, which suggests that arsenic exposure could disrupt the homeostasis of Th1/Th2/Th17/Treg to induce immune inflammatory responses. Those are consistent with partial conclusions that arsenic altered the level of immune cytokines in preschool children [37] as well as male workers exposed to arsenic in Bangladesh [38]. In addition, researchers also found that arsenic inhibited the cellular immunity by changing the expression of Th1-related cytokines but not affecting Th2 in preschool children [33]. Another in vitro study found that low-dose arsenic (0.25–2 *μ*mol) exposure reduced the secretion of IFN-*γ* without influencing IL-4 and IL-13 [39]. It is reported that IL-23 could affect the numbers and their capacity to secrete IL-10 of Treg cells [40]. In our study, it was found that the ratio of IFN-*γ* to IL-13 increased in the liver exposed to arsenic as well as decreased in the arsenic-treated groups except the 25 mg/L NaAsO_2_ group with 1 month in the kidney, which indicated that the effect of arsenic on Th1 is greater than Th2 in the liver and a dominate Th2 in the kidney. The difference in results between the liver and kidney may be due to tissue diversity. In patients with coal-burning arsenic poisoning, the imbalance of Th17 and Tregs in peripheral blood mononuclear cells also appeared, characterized by the high level of Th17, and corresponding cytokines as well as decreased Tregs and IL-10 [41]. Our results found that the ratio of IL-23 to IL-10 markedly increased in the liver and kidney, which suggested that the homeostasis between Th17 and Tregs was disrupted as well as prone to Th17 in the liver and kidney. In summary, arsenic undermined the dynamic balance of T lymphocyte subpopulation, which may be closely related to arsenic-induced immune-inflammation, immunosuppression, and the occurrence and development of cancer.

Recently, MAPKs, comprising of ERK1/2, JNK ,and P38, have been reported to regulate innate and adaptive immune response as well as mediate the expression of inflammatory cytokines including COX-2, TNF-*α*, and IL-1*β*, through the regulation of transcription factors NF-*κ*B and activator protein 1 (AP-1) [42, 43]. In addition, the MAPK pathway is highly associated with cancers in human, and NF-*κ*B is a proinflammatory transcription factor and may be involved in many physiological and pathological processes, including proliferation, apoptosis, and oncogenesis [20]. It has been reported that NF-*κ*B and MAPK activation could participate in CD4^+^ T cell subpopulation Th17 and Treg cell differentiation [44]. Kim et al. found that 0.5 *μ*M As_2_O_3_ significantly increased the P38MAPK protein expression in BALB/C 3T3 cells (a model widely used to study cancer development) [45]. In our results, all three members of MAPKs, namely, phospho-ERK, phospho-JNK ,and phospho-P38, as well as downstream target NF-*κ*B were upregulated remarkably in arsenic-treated mice. This implied the persistent inductive effect on MAPK and the NF-*κ*B pathway in the liver and kidney after the subchronic arsenic exposure.

Generally, Nrf2 is a transcription factor that regulates the expression of NAD(P)H quinone oxidoreductase 1 (NQO1), heme oxygenase 1 (HMOX1), glutamate-cysteine ligase (GCL), and glutathione S transferases (GST) under oxidative stress, which could then counteract the oxidative damage of the metalloid [46]. Besides its antioxidant functions, many recent studies have demonstrated that Nrf2 could regulate the expression of immune molecules and inflammatory factors and then play an important role in immune and inflammatory diseases [47]. In terms of cancer, Nrf2 has emerged as somewhat a double-edged sword, because it is not only involved in cancer development but also in cancer treatment [48]. In our result, subchronic arsenic exposure has been shown to potently upregulate the expression of hepatic and renal Nrf2 and its downstream genes GST and GCLM. Li et al. found that acute arsenic exposure resulted in a clear increase of Nrf2, GST, and GCLC protein in both the liver and kidney in vivo [49]. Activation of the Nrf2 pathway by acute arsenic is regarded as a biological defense mechanism and helpful response. However, constitutive Nrf2 activation is an excessive response, which is involved in increasing cancer chemoresistance and enhancing tumor cell growth [50]. It is therefore suggested that one of the main mechanisms involved in the hepatic and nephritic immune-inflammatory abnormalities of arsenic is associated with activating the Nrf2 pathway.

In conclusion, we demonstrated that subchronic arsenic exposure may induce hepatonephric toxicity through the modulation of inflammation and CD4^+^ T cell differentiation, which may be regulated through the activation of the MAPK/NF-*κ*B and Nrf2 pathways.

## Figures and Tables

**Table 1:** Average daily arsenic intake in drinking water, the concentrations of total arsenic (T-As, ng As/g tissue) of the liver and kidney, the body weight and liver and kidney weights in control and different experimental mice.

Experimental groups	Duration (months)	Dose (mg/L)	Calculated average daily arsenic intake (mg NaAsO_2_/kg body weight/day)	T-As in liver (ng/g)	T-As in kidney (ng/g)	Body weight (g)	Liver weight (g)	Kidney weight (g)
Group 1	1	0	0	<LD	<LD	31.85 ± 1.08	1.44 ± 0.20	0.28 ± 0.02
Group 2	1	25	4.06 ± 0.90	93.97 ± 36.64	44.2 ± 3.18	31.64 ± 2.63	1.37 ± 0.16	0.29 ± 0.03
Group 3	1	50	6.47 ± 2.31	123.58 ± 22.65	64.28 ± 8.26	32.04 ± 1.56	1.35 ± 0.20∗	0.30 ± 0.03
Group 4	3	0	0	<LD	<LD	38.32 ± 1.86	1.36 ± 0.17	0.29 ± 0.03
Group 5	3	25	3.41 ± 0.23	213.29 ± 86.28	37.92 ± 6.16	36.05 ± 2.00	1.16 ± 0.18∗	0.29 ± 0.04
Group 6	3	50	6.47 ± 0.33	229.95 ± 21.04	66.36 ± 20.22	38.52 ± 0.89	1.13 ± 0.11∗	0.31 ± 0.03

Mice were treated with 25 and 50 mg/L NaAsO_2_ by drinking water for 1 and 3 months, and total arsenic (T-As) levels of the liver and kidney were determined by the high-performance liquid chromatography-hydride generation-atomic fluorescence spectrometry (HPLC-HG-AFS) method, as described in Materials and Methods. Results were expressed as mean ± SD (*n* = 3). The limit of detection (LD) for T-As was 1 *μ*g/L. ^∗^Denoted *P* < 0.05 compared with control mice.

## Data Availability

No data were used to support this study.

## Abbreviations

ALT: Alanine aminotransferase
AST: Aspartate aminotransferase
AP1: Activator protein 1
COX-2: Cyclooxygenase 2
ERK1/2: Extracellular-signal-regulated kinases1/2
GCL: Glutamate-cysteine ligase
GST: Glutathione S transferase
HMOX1: Heme oxygenase 1
IL-1*β*: Interleukin 1*β*
JNK: C-Jun N-terminal kinases
MPO: Myeloperoxidase
MAPK: Mitogen-activated protein kinase
NF-*κ*B: Nuclear factor kappa B
Nrf2: Nuclear factor E2-related factor 2
NQO1: NAD(P)H quinone oxidoreductase 1
ROS: Reactive oxygen species
TNF- *α*: Tumor necrosis factor-*α*
T-As: Total arsenic
Th1: T-helper 1
Treg: Regulatory T cells.

## Additional Points

*Highlights*. Arsenic induced persistent inflammatory responses in the liver and kidney. Arsenic damaged Th1/Th2/Th17/Treg balance in the liver and kidney. Arsenic activated the MAPK/NF-*κ*B and Nrf2 pathway continuously in the liver and kidney.

## References

[1] Ferrario D., Gribaldo L., Hartung T. (2016). Arsenic exposure and immunotoxicity: a review including the possible influence of age and sex. *Current Environmental Health Reports*.

[2] Naujokas M. F., Anderson B., Ahsan H. (2013). The broad scope of health effects from chronic arsenic exposure: update on a worldwide public health problem. *Environmental Health Perspectives*.

[3] Dutta K., Prasad P., Sinha D. (2015). Chronic low level arsenic exposure evokes inflammatory responses and DNA damage. *International Journal of Hygiene and Environmental Health*.

[4] Tsao D. A., Tseng W. C., Chang H. R. (2017). RKIP expression of liver and kidney after arsenic exposure. *Environmental Toxicology*.

[5] Costa M. (2019). Review of arsenic toxicity, speciation and polyadenylation of canonical histones. *Toxicology and Applied Pharmacology*.

[6] Flora S. J., Mittal M., Pachauri V., Dwivedi N. (2012). A possible mechanism for combined arsenic and fluoride induced cellular and DNA damage in mice. *Metallomics*.

[7] Adil M. K., Kandhare A. D., Visnagri A., Bodhankar S. L. (2015). Naringin ameliorates sodium arsenite-induced renal and hepatic toxicity in rats: decisive role of KIM-1, Caspase-3, TGF-*β*, and TNF-*α*. *Renal Failure*.

[8] Dong C., Helper T. (2017). Helper T Cells and cancer-associated inflammation: a new direction for immunotherapy?. *Journal of Interferon & Cytokine Research*.

[9] Gera R., Singh V., Mitra S. (2017). Arsenic exposure impels CD4 commitment in thymus and suppress T cell cytokine secretion by increasing regulatory T cells. *Scientific Reports*.

[10] Duan X., Gao S., Li J. (2017). Acute arsenic exposure induces inflammatory responses and CD4^+^ T cell subpopulations differentiation in spleen and thymus with the involvement of MAPK, NF-kB, and Nrf 2. *Molecular Immunology*.

[11] Nadeau K. C., Li Z., Farzan S. (2014). In utero arsenic exposure and fetal immune repertoire in a US pregnancy cohort. *Clinical Immunology*.

[12] Dong C. (2021). Cytokine regulation and function in T cells. *Annual Review of Immunology*.

[13] Banerjee S., Mitra T., Purohit G. K., Mohanty S., Mohanty B. P. (2015). Immunomodulatory effect of arsenic on cytokine and HSP gene expression in Labeo rohita fingerlings. *Fish & Shellfish Immunology*.

[14] Li C., Zhang J., Wang W., Wang H., Zhang Y., Zhang Z. (2019). Arsenic trioxide improves Treg and Th17 balance by modulating STAT3 in treatment-naïve rheumatoid arthritis patients. *International Immunopharmacology*.

[15] Li N., Hua J. (2017). Immune cells in liver regeneration. *Oncotarget*.

[16] Denecke C., Tullius S. G. (2014). Impact des lesions d'ischemie-reperfusion sur la reponse immunitaire innee et acquise. *Progrès en Urologie*.

[17] Singh N., Baby D., Rajguru J. P., Patil P. B., Thakkannavar S. S., Pujari V. B. (2019). Inflammation and cancer. *Annals of African Medicine*.

[18] Huang H. W., Lee C. H., Yu H. S. (2019). Arsenic-induced carcinogenesis and immune dysregulation. *International Journal of Environmental Research and Public Health*.

[19] Das S., Joardar S., Manna P. (2018). Carnosic acid, a natural diterpene, attenuates arsenic-induced hepatotoxicity via reducing oxidative stress, MAPK activation, and apoptotic cell death pathway. *Oxidative Medicine and Cellular Longevity*.

[20] Peng L., Hu C., Zhang C., Lu Y., Man S., Ma L. (2020). Anti-cancer activity of *Conyza blinii* saponin against cervical carcinoma through MAPK/TGF- *β*/Nrf2 signaling pathways. *Journal of Ethnopharmacology*.

[21] Afolabi O. K., Wusu A. D., Ogunrinola O. O. (2015). Arsenic-induced dyslipidemia in male albino rats: comparison between trivalent and pentavalent inorganic arsenic in drinking water. *BMC Pharmacology and Toxicology*.

[22] Sun X., Li J., Zhao H. (2018). Synergistic effect of copper and arsenic upon oxidative stress, inflammation and autophagy alterations in brain tissues of Gallus gallus. *Journal of Inorganic Biochemistry*.

[23] Li J., Guo Y., Duan X., Li B. (2020). Tissue- and region-specific accumulation of arsenic species, especially in the brain of mice, after long-term arsenite exposure in drinking water. *Biological Trace Element Research*.

[24] Kleiner D. E., Brunt E. M., van Natta M. (2005). Design and validation of a histological scoring system for nonalcoholic fatty liver disease. *Hepatology*.

[25] Ghatak S., Biswas A., Dhali G. K., Chowdhury A., Boyer J. L., Santra A. (2011). Oxidative stress and hepatic stellate cell activation are key events in arsenic induced liver fibrosis in mice. *Toxicology and Applied Pharmacology*.

[26] Xu Y. Y., Zeng Q. B., Yao M. L., Yu C., Li J., Zhang A. H. (2016). A possible new mechanism and drug intervention for kidney damage due to arsenic poisoning in rats. *Toxicology Research*.

[27] Adeyemi O. S., Meyakno E., Akanji M. A. (2017). Inhibition of Kupffer cell functions modulates arsenic intoxication in Wistar rats. *General Physiology and Biophysics*.

[28] Kotyzová D., Bludovská M., Eybl V. (2013). Differential influences of various arsenic compounds on antioxidant defense system in liver and kidney of rats. *Environmental Toxicology and Pharmacology*.

[29] Wang Y., Zhao H., Shao Y., Liu J., Li J., Xing M. (2017). Copper or/and arsenic induce oxidative stress-cascaded, nuclear factor kappa B-dependent inflammation and immune imbalance, trigging heat shock response in the kidney of chicken. *Oncotarget*.

[30] Elinav E., Nowarski R., Thaiss C. A., Hu B., Jin C., Flavell R. A. (2013). Inflammation-induced cancer: crosstalk between tumours, immune cells and microorganisms. *Nature Reviews. Cancer*.

[31] Zhang K., Zhao P., Guo G. (2016). Arsenic trioxide attenuates NF-*κ*B and cytokine mRNA levels in the livers of cocks. *Biological Trace Element Research*.

[32] Liu J., Wang Y., Zhao H. (2020). Arsenic (III) or/and copper (II) exposure induce immunotoxicity through trigger oxidative stress, inflammation and immune imbalance in the bursa of chicken. *Ecotoxicology and Environmental Safety*.

[33] Abdul K. S., Jayasinghe S. S., Chandana E. P., Jayasumana C., De Silva P. M. (2015). Arsenic and human health effects: a review. *Environmental Toxicology and Pharmacology*.

[34] Yan J., Smyth M. J., Teng M. W. L. (2018). Interleukin (IL)-12 and IL-23 and their conflicting roles in cancer. *Cold Spring Harbor Perspectives in Biology*.

[35] Liang P., Peng S., Zhang M., Ma Y., Zhen X., Li H. (2017). Huai Qi Huang corrects the balance of Th1/Th2 and Treg/Th17 in an ovalbumin-induced asthma mouse model. *Bioscience Reports*.

[36] Sifnaios E., Mastorakos G., Psarra K. (2019). Gestational diabetes and T-cell (Th1/Th2/Th17/Treg) immune profile. *In Vivo*.

[37] Zhang Y., Huo X., Lu X., Zeng Z., Faas M. M., Xu X. (2020). Exposure to multiple heavy metals associate with aberrant immune homeostasis and inflammatory activation in preschool children. *Chemosphere*.

[38] Parvez F., Lauer F. T., Factor-Litvak P. (2019). Assessment of arsenic and polycyclic aromatic hydrocarbon (PAH) exposures on immune function among males in Bangladesh. *PLoS One*.

[39] Morzadec C., Bouezzedine F., Macoch M., Fardel O., Vernhet L. (2012). Inorganic arsenic impairs proliferation and cytokine expression in human primary T lymphocytes. *Toxicology*.

[40] Stewart C. A., Trinchieri G. (2009). Reinforcing suppression using regulators: a new link between STAT3, IL-23, and Tregs in tumor immunosuppression. *Cancer Cell*.

[41] Xia S., Sun Q., Zou Z. (2020). Ginkgo biloba extract attenuates the disruption of pro-and anti-inflammatory T-cell balance in peripheral blood of arsenicosis patients. *International Journal of Biological Sciences*.

[42] Medeiros M. C., Frasnelli S. C., Bastos A. D., Orrico S. R., Rossa Junior C. (2014). Modulation of cell proliferation, survival and gene expression by RAGE and TLR signaling in cells of the innate and adaptive immune response: role of p 38 MAPK and NF-KB. *Journal of Applied Oral Science*.

[43] Lu H., Wang B., Cui N., Zhang Y. (2018). Artesunate suppresses oxidative and inflammatory processes by activating Nrf2 and ROS-dependent p38 MAPK and protects against cerebral ischemia-reperfusion injury. *Molecular Medicine Reports*.

[44] Lim S. M., Jeong J. J., Kang G. D., Kim K. A., Choi H. S., Kim D. H. (2015). Timosaponin AIII and its metabolite sarsasapogenin ameliorate colitis in mice by inhibiting NF-*κ*B and MAPK activation and restoring Th17/Treg cell balance. *International Immunopharmacology*.

[45] Kim H. G., Shi C., Bode A., Dong Z. (2016). p38*α* MAPK is required for arsenic-induced cell transformation. *Molecular Carcinogenesis*.

[46] Xu M., Niu Q., Hu Y., Feng G., Wang H., Li S. (2019). Proanthocyanidins antagonize arsenic-induced oxidative damage and promote arsenic methylation through activation of the Nrf2 signaling pathway. *Oxidative Medicine and Cellular Longevity*.

[47] Kim J., Surh Y. J. (2009). The role of Nrf2 in cellular innate immune response to inflammatory injury. *Toxicology Research*.

[48] Rojo De La Vega M., Chapman E., Zhang D. D. (2018). NRF2 and the hallmarks of cancer. *Cancer Cell*.

[49] Li J., Duan X., Dong D. (2015). Hepatic and nephric NRF2 pathway up-regulation, an early antioxidant response, in acute arsenic-exposed mice. *International Journal of Environmental Research and Public Health*.

[50] Kansanen E., Kuosmanen S. M., Leinonen H., Levonen A. L. (2013). The Keap1-Nrf2 pathway: mechanisms of activation and dysregulation in cancer. *Redox Biology*.

